# A qualitative study examining healthcare managers and providers’ perspectives on participating in primary care implementation research

**DOI:** 10.1186/s12913-016-1577-1

**Published:** 2016-07-29

**Authors:** Lisa A. Wozniak, Allison Soprovich, Sandra Rees, Steven T. Johnson, Sumit R. Majumdar, Jeffrey A. Johnson

**Affiliations:** 1 2-040 Li Ka Shing Centre for Health Research Innovation, School of Public Health, University of Alberta, Edmonton, T6G 2G3 Canada; 2 Faculty of Health Disciplines, Athabasca University, Athabasca, AB Canada; 3 5-112 Clinical Sciences, Department of Medicine, University of Alberta, Edmonton, T6G 2G3 Canada

**Keywords:** Primary care, Implementation research, Qualitative study, Healthcare provider perspectives, Experimental study design

## Abstract

**Background:**

Primary care reforms should be supported by high-quality evidence across the entire life cycle of research. Front-line healthcare providers play an increasing role in implementation research. We recently evaluated two interventions for people with type 2 diabetes (T2D) in partnership with four Primary Care Networks (PCNs) in Alberta, Canada. Here, we report healthcare professionals perspectives on participating in primary care implementation research.

**Methods:**

Guided by the RE-AIM framework, we collected qualitative data before, during, and after both interventions. We conducted 34 in-person or telephone interviews with 17 individual PCN professionals. We used content analysis to identify emerging codes and concepts.

**Results:**

Two major themes emerged from the data. First, healthcare managers were eager to conduct implementation research in a primary care setting. Second, regardless of willingness to conduct research, there were challenges to implementing experimental study designs for both interventions. PCN professionals presumed the interventions were better than usual care, expressed role conflict, and reported administrative burdens related to research participation. Perceptions of patient vulnerability and an obligation to intervene exacerbated these issues.

**Conclusions:**

Healthcare professionals with limited practical research experience might not foresee the challenges in implementing experimental study designs in primary care settings to generate high-quality evidence. These issues are intensified when healthcare professionals perceive target patient populations as vulnerable and in need of intervention based on the presenting illness. Possible solutions include further research training, involving healthcare professionals in study design development, and using non-clinical staff to conduct research activities, particularly among acutely unwell patient populations.

## Background

There is a need to transform primary care, and one of the most important methods relates to conducting rigorous implementation research [1]. Increasingly, front-line healthcare providers are participating in implementation research [2] and can influence the quality of trials and the evidence generated [3]. However, we know little about their perspectives on participating in primary care research. Most studies focus on healthcare providers’ views of participating in traditional randomized controlled trials, typically in academic or tertiary care settings [2–8], and primary care related implementation settings remain under-studied.

We recently evaluated the implementation of two interventions for people with type 2 diabetes (T2D) in partnership with four Primary Care Networks (PCNs) in Alberta, Canada. PCNs are similar to the medical home model in the US [9–11]. They are a network of physicians and allied healthcare providers who provide comprehensive primary care tailored to local populations. PCN healthcare professionals implemented the two interventions, supported by our research team. Healthy Eating and Active Living for Diabetes in Primary Care Networks (HEALD) was a 6-month, exercise-specialist led, pedometer-based walking intervention [12]. TeamCare was a 12-month collaborative care intervention led by a nurse care manager who coordinated the care of patients with T2D and depressive symptoms [13]. Both interventions were previously demonstrated to be efficacious in controlled studies [14–16], and proved effective when implemented in Alberta’s PCN settings [17, 18]. As part of our detailed mixed-methods evaluation plans, we employed the RE-AIM framework [19–21] a priori to evaluate the implementation of both interventions [12, 13, 22]. Herein, we describe PCN healthcare managers and providers’ perspectives on participating in primary care implementation research.

## Methods

### Setting: primary care networks

We implemented HEALD and TeamCare in partnership with four non-metro PCNs (i.e., serving an area of less than 50,000 people) between 2010 and 2013. While unique in structure, all four PCNs shared a chronic disease management mandate making them an ideal environment to adopt the interventions. Collectively, the four PCNs represented 140 family physicians serving approximately 10,000 patients with T2D. Healthcare managers and providers at the four PCNs had varying levels of experience participating in research, ranging from no experience to collaborating with research teams responsible for implementing studies in the PCN setting. We promoted PCN ownership of the research by aligning with their organizational values (e.g., chronic disease mandate) and activities (e.g., existing diabetes, depression, and lifestyle counseling programs) [23], tailoring protocols to each PCN (e.g., providing strategies to achieve physician support, patient tracking template, sample patient invitation letter) [24, 25], and providing financial resources to undertake the interventions.

### Research training

PCN managers recruited or seconded healthcare providers to deliver the interventions (i.e., exercise specialists for HEALD and nurse case managers for TeamCare). The healthcare providers were responsible for patient recruitment, screening, obtaining informed consent, patient allocation to study arms, and data collection and management. To ensure the integrity of the research designs across sites, healthcare providers received comprehensive resources and extensive training. This included detailed project manuals, telephone screening scripts, information letters and consent forms, data collection forms and surveys, and instructions on data entry and management (Table 1). We provided on-site training and detailing sessions on informed consent, data collection and management, and performing clinical and physical measures. In addition, we provided regular quality assurance feedback and on-going support through site visits, telephone and email communications (Table 2).

**Table 1:** Research protocols provided to PCN healthcare providers, including documents, processes, and systems

Documents/processes/systems
➢ Project background, including development of the interventions (HEALD and TeamCare)
➢ Contact lists (research team, participating PCN staff across sites)
➢ Protocol to achieve member physician support
➢ On/Off study design timetable/schedule
➢ Patient recruitment algorithm/study flow, including: ▪ Protocol for non-response to patient invitation letter
➢ Patient recruitment and tracking system (Excel)
➢ Short screening survey package, including: ▪ Sample patient invitation letter from PCN ▪ Information letter
➢ Eligibility & incentives, including: ▪ Inclusion/Exclusion criteria ▪ Incentive
➢ Telephone screening script and protocol
➢ Frequently Asked Questions
➢ Checklist for packages sent to participants (HEALD specific)
➢ Data collection matrix
➢ Clinical protocols/instructions for intervention and active control groups, including: ▪ Informed consent ▪ Data collection of clinical & physical measures ▪ Discharge or end of study
➢ Information letters & consent forms
➢ Data collection forms & surveys
➢ Instructions for data entry & management

**Table 2:** Summary of training activities/supports provided to PCN healthcare providers responsible for implementing the study designs

Training activities & supports by research team	Members of the PCN team
▪ Individual detailing sessions on: ▪ Obtaining informed consent ▪ Data collection and documentation (e.g., assigning study IDs) ▪ Data management (e.g., proper secure storage of patient files) ▪ Patient recruitment algorithms/study flow, including mail out of screening survey packages and reminder postcards ▪ Eligibility criteria ▪ Telephone screening script & protocol ▪ Preforming clinical and physical measures	▪ Exercise specialists and care managers ▪ Administrative personnel
▪ On-site training on patient recruitment & tracking system (e.g., Access database)	▪ Exercise specialists and care managers ▪ Administrative personnel
▪ On-site Point of Care training	▪ Exercise specialists and care managers
▪ On-going support by research team via regular in-person PCN site visits, telephone, electronic mail, and quarterly bulletin	▪ Exercise specialists and care managers ▪ Administrative personnel

### Study design

We described the overall evaluation [22] and the study design for HEALD [12] and TeamCare [13] elsewhere. To recruit for both interventions, healthcare providers telephoned potential participants to establish willingness to participate and confirm eligibility. Once confirmed, healthcare providers allocated eligible patients using a controlled “on-off” time-series method [26, 27]. This ‘alternation’ form of allocation is credible for health care quality improvement studies, and resulted in no imbalances or detectable biases between the intervention and control groups [27]. Control group patients received usual care.

### Data collection

Guided by the RE-AIM framework [22], we collected qualitative data at baseline, midpoint, and post-intervention for both interventions. The primary source of data was interviews with healthcare managers (i.e., executive directors and managers of chronic disease programming) and providers (i.e., nurse care managers and exercise specialists) across the four PCNs who were involved in the interventions. Using purposeful sampling, we invited via email all healthcare managers and providers involved to participate in semi-structured interviews at three time points (i.e., baseline, midpoint, and post-intervention), as appropriate, using interview guides (Table 3). All invited participants agreed to interviews. Participants were informed of the research goals. One researcher (LW) conducted and digitally recorded 34 interviews with 17 individuals, either in-person at the PCN offices or by telephone. Interviews were 30 to 120 min in length and were transcribed verbatim by an independent transcriptionist and verified for accuracy. The Health Ethics Research Board of the University of Alberta deemed this qualitative component of the intervention studies exempt from review [22].

**Table 3:** Selected questions from interview guides by timeline and key informant groups

Timeline	Key informant group(s)^a^	Selected interview questions^b^
Baseline	ED & CDM	Why did your PCN decide to implement and deliver HEALD and TeamCare? In other words, why did you think the interventions were a good fit for your PCN?
		Describe why you provided a rating of *X* ^c^ to describe the level of commitment of the PCN leadership (i.e., PCN Board and/or Management) to the HEALD and TeamCare interventions.
		At this time, do you believe that PCN staff is capable of implementing HEALD and TeamCare? Why?
		At this time, do you believe HEALD and TeamCare are appropriate for PCN staff, patients, and/or PCN member physicians? Why?
		What are the anticipated benefits of HEALD and TeamCare, if any, for staff? What are the anticipated disadvantages, if any, to staff?
		Do you have any other comments about HEALD or TeamCare?
Midpoint	ED, CDM, CM, & ES	To date, what were the challenges or barriers in getting HEALD and TeamCare up and running? How were these challenges or barriers addressed?
		To date, what has worked well in getting HEALD and TeamCare up and running?
		Looking back, what would you have done differently or do you think should have been done differently.
		What advice or recommendations would you give to someone else in your position that is about to implement HEALD and TeamCare into their organization?
		What assumptions did you make about HEALD and TeamCare (e.g., your role, interventions, how it was going to work)?
		In your opinion, are HEALD and TeamCare sustainable? If yes, how do you see this being achieved? If no, why?
		Do you have any other comments about HEALD or TeamCare?
Post-Intervention	ED, CDM, CM, & ES	During your midpoint interview, we focused mainly on implementation of TeamCare. Do you have anything else to add regarding what worked well, what didn’t work well, and suggestions for improvement?
		Thinking back to when we first asked you to be involved, what assumptions did you make about HEALD and TeamCare (e.g., your role, the intervention, how it was going to work)?
		In your opinion, should the PCN continue to use this model of care (i.e., HEALD and TeamCare)? Why or why not?
	ED & CDM	What components, if any, of HEALD and TeamCare will be sustained in your PCN? Describe how each component will be sustained.
		What components, if any, of HEALD or TeamCare will not be sustained? Describe the barriers to sustaining these components. What would make it sustainable?
	CM & ES	Tell me about your (job) satisfaction in this role compared to your previous roles.
		Based on your experience, would you consider doing this role again? Why or why not?
		What will you take, if anything, from your experience with HEALD and TeamCare into your future work/roles?
	ED, CDM, ES, & CM	Do you have any other comments about HEALD or TeamCare?

*ED* executive directors who were responsible for supervising the CDM and ES

*CDM* chronic disease managers who were responsible for supervising the CDM and ES

*CM* Nurse care managers who were responsible for implementing the research and intervention activities for the TeamCare intervention

*ES* exercise specialists who were responsible for implementing the research and intervention activities for the HEALD intervention

*PCN* primary care network

^a^Key informant groups were interviewed based on timing of the interventions and relevance to their role. For example, exercise specialists and care managers were not asked baseline interview questions because they were not involved in the decision to implement the interventions at each PCN. In addition, exercise specialists were not asked to comment on the TeamCare intervention because they were not involved in its implementation

^b^ Key informant groups were asked each interview question separately for the HEALD and TeamCare interventions

^c^ The rating was elicited through the following question asked in the usual care checklist: Considering all the competing priorities your PCN has, what is the level of commitment of the PCN leadership (i.e., PCN Board and/or Management) to the TeamCare-PCN intervention on a scale of 1 to 10, where 1 is “not a priority at all” and 10 is “highest priority of all”?

### Data analysis

We used an inductive, content analysis approach to identify emerging codes and concepts related to participating in implementation research in the primary care setting, as this is the most appropriate analytic technique for an exploratory qualitative study [28, 29]. Emerging codes and concepts were discussed during formal research team meetings and discrepancies resolved by consensus. Data saturation was achieved through interviewing all key informants involved in implementing the research and intervention activities across the participating PCNs. We used several verification strategies [30], including methodological coherence, appropriate sampling, and concurrent data collection and analysis [30]. We participated in prolonged engagement with the PCNs [29, 31] (e.g., developed and maintained a 4-year partnership), member checking [31], and peer debriefing [28]. To ensure participant and PCN confidentiality, we used study codes for highlighted quotes reflecting respondents (i.e., managers or providers) and intervention (i.e., “HD” for HEALD and “TC” for TeamCare). Data were managed using Nvivo 10 (Burlington, MA, QSR International (Americas) Inc.) [32].

## Results

Two major themes emerged regarding participating in primary care implementation research. First, healthcare managers were eager to participate in primary care implementation research. Second, regardless of the willingness to participate in research, there were challenges implementing experimental study designs and protocols for both interventions. Healthcare managers and providers presumed the interventions were better than usual care, experienced role conflict, and reported participating in research as administratively burdensome. We found that perceptions of the vulnerability of the patient population and duty to intervene based on severity of the presenting illness exacerbated these issues. We provide illustrative quotes for each theme and additional supporting quotations in Table 4.

**Table 4:** Supporting quotations by themes and key findings

Key findings	Supporting quotation(s)
Theme 1: Eager to participate in primary care implementation research	
▪ Supportive of implementation research in primary care setting to determine effectiveness of interventions and to use evidence to inform decision-making	*I mean it’s participating in research, definitely. Because we want to prove what we’re doing is working, right? Or else, why are we here? (PCN manager-HD/TC).*
	*So there are millions of dollars being spent and very little actually researching whether it’s effective or if there are better ways to do it… (PCN manager-HD/TC).*
	*Looking at more informing our future business planning…We have two years to evaluate how effective this is and it’s a really easy sell for the Board after the fact to say, look, this is the effect of it (PCN manager-HD/TC).*
	*I think that the results are going to be important in terms of trying to actually put some dedicated funding into the continuation of it. Often with research, we’re there with the resources, but then once the research project finishes, the reserves are spent. So really making it a continued priority in the PCN, to continue putting resources towards these two projects…with the business planning process (PCN manager-HD/TC)*
▪ Implementation research perceived as external to PCN (i.e., university-based)	*Right now it’s a research study. But I think as [TeamCare] becomes more of a way of doing business, more physicians would come onboard. Because it is the way we practice then (PCN manager-TC).*
	*I mean even though I was housed within the PCN, I didn’t always necessarily feel like I got the support from the other PCN team members… But I think it was easier rather than trying to understand [HEALD] and continuing to fail, I think it was almost easier for them to just pretend, oh they didn’t exist, right? Like pretend that “HEALD, oh that’s the research study and they’re doing their own thing”, rather than, “Okay how can we work as a team and how can – how can we help you?” (PCN provider-HD).*
	*As far as [the exercise specialist], that was completely outside of anything we were doing [at the PCN]. So she was really working with you guys. It didn’t affect us at all (PCN manager-HD).*
Theme 2: Challenges to conducting primary care implementation research	
➢ Interventions presumed better than usual care ▪ Study design is not appropriate for this stage of research	*Honestly, the on-off [design] has been one of the major challenges. And I know in a pure research, laboratory, very scientific methods, it makes abundant sense to do that because that is the gold standard, right? But in reality, I think that’s why you see so few people-related studies that are having an [intervention and active-control] group – unless it’s like a pharmaceutical double blind (PCN manager-HD/TC).*
	*The on-off design? What I would do differently primarily would be… to my perspective, this was an implementation study and so the reality is, we have existing services. So the [control group] would be reasonable to use that with our existing [PCN] services…Rather [than] coming in with the attitude of “You are not effectively managing patients, let us show you how to do it, and so we’re going to, on our island, tell you how things should be done” – I feel that that was a really unfortunate approach. Because the existing services are very similar, at least within our PCN – very, very similar… Rather than saying, you know, “on-off - we just don’t take care of these people”. [Instead, for the active-control arm], this is usual care, using our mental health coordinators, and wouldn’t it be nice if the results are actually equally as positive (PCN provider-TC).*
▪ Poor patient engagement and retention of control group patients, specific to TeamCare	*The challenge I’m having now is the [active-control group] people, they’re just not as engaged, of course. And to get them to come back in for their six-month [data collection appointment] is very challenging, you know? And we’ve had a few that have dropped out… But right now, my major challenge is to get those people in who are due for their six months who are [in the active-control group]. Yeah, it’s a lot of phone calls, you know, and follow-up with them and it is a challenge to get them. It’s almost like, “what’s in it for me”, you know? (PCN provider-TC).*
	*[The care manager] and I were just talking about it, not too long ago, saying, well that [active-control] group – what’s the incentive for them to come back in six months? You know? And that’s probably where you lose a lot of people, the attrition is that “what’s the point of coming back? They didn’t do anything the first time for me, what are they going to do the second time”? (PCN manager-TC).*
	*Having an unblinded study where there’s the [intervention] group and the [active-control] group and then this [active-control] group doesn’t really get anything from us other than having to fill out questionnaires. So I think that in itself was a bit of a difficulty to keep people in the study, difficult to keep them interested if they were proposed that, “Okay, this is the consent. By the way, you’re in the [active-control] group”, and then they’ll be like “Well what’s in it for me?” Right? (PCN provider-TC).*
▪ Difficulty referring control group patients to usual care, specific to TeamCare	*We have to go back to the physician and the physician has to refer them [to the PCN]. And if that physician isn’t engaged with the PCN and what we have to offer, that patient doesn’t get seen, even though they may score high on their testing. So that’s been very difficult for us. Hands off (PCN manager-TC).*
➢ Role conflict ▪ Experimental study design conflicts with professional commitment to provide care	*Yeah, ‘cause it’s against what we – like you’re here to help people and it kind of goes against your grain to not be able to say “Well we could help you with that”, or “the [PCN] program here is great” or [city name] mental health, here’s their number… because we’ve had other people who’ve done really well with some small changes to their medication [in TeamCare]. So you want that for everyone (PCN provider-TC).*
	*I think one of the things that we’ve talked a little bit about here as one of the challenges is… it’s sort of the [intervention] and [active-control] group idea, right? Like ‘cause obviously it’s been really hard for the nurses to say, “Well you know, you’re demonstrating all these symptoms and maybe you’re not managing so well with your diabetes but you can’t be part of the program”. And so that’s been a bit of a struggle for us, for sure… I know it was hard for the nurses to say, “Sorry” (PCN manager-HD/TC).*
	*So that way you wouldn’t have this group of people that you feel some of them have been the people that needed it the worst, you know? Looking at their PHQ scores and talking with them even just a brief amount of time that you talk. Actually some of them I’ve spent quite a bit of time talking to because they’re a 20 [on the PHQ]. You can’t really offer them everything. You think, “Oh I’d really like to follow up this individual” and you can’t do it. So it’s kind of annoying – not right. Morally not right for them. So that’s one thing I would change (PCN provider-TC).*
▪ Discomfort with experimental study design (e.g., unethical or immoral), specific to TeamCare	*Well I still have a hard time with the [active-control] group. Like I have to say like yesterday I had a client who scored 21 [on the PHQ]. Like I would have maybe liked to see this as a pilot project and then evaluate rather than having the intervention and non-intervention. I don’t know. It just feels sometimes unethical to bring people in – especially if their score is high. Like if they’re ten – but this fellow yesterday, just as an example, he was 21, his A1c was 11.1, his lipids were all elevated, blood pressure – and then I had to say, “Hmmm. Well thanks for coming – thanks for coming out”. So that feels unethical to me somehow ‘cause we know we could offer something more. So … I have a hard time with that (PCN provider-TC).*
	*It’s an ethical clinical issue, yes. There’s research protocols…that will always be less significant than somebody’s physical safety. And that’s not a negotiable for me…compared to “is someone appropriately being treated for the risk of suicide”…The issue was “here’s the bigger picture” – patient safety should, in my mind as a clinician, have to come [first] – and it didn’t feel like it was. And that’s hard to do as a nurse. Especially as a legally - you know, as the legal expectation for myself that I would not compromise in any other setting. (PCN provider-TC).*
	*That way you wouldn’t have this group of people that you feel that some of them have been the people that needed [the intervention] the worst. Like looking at their PHQ scores and talking with them even just a brief amount of time - actually some of them I’ve spent quite a bit of time talking – because they’re a twenty [score on PHQ]… you can’t really offer them everything. You think, “Oh I’d really like to follow up this individual” and you can’t do it. So it’s kind of annoying, not right. Morally not right for them (PCN provider-TC).*
	*We’ve all had a really hard time with those patients in the [active-control] group. And that’s so hard on our staff. That has been a very hard thing because we have a mental health liaison that would be the natural fit. That’s what we would naturally do. It is. But we can’t tell [patients] that. We have to go back to the physician and the physician has to refer them. And if that physician isn’t engaged with the PCN and what we have to offer, that patient doesn’t get seen, even though they may score high on their [PHQ]. That’s been very difficult for us. Hands off…And, what about ethics? Where does ethics play in something like that? Because, really, that’s not ethical (PCN manager-TC).*
▪ Recommendations to modify the study design	*Well, I guess my surprise was the on-off [design], right? I had some concerns about that initially. And I talked to [the Research Program Manager] about it and, I think as a team, you likely had some concerns about that as well. But we’re committed, that’s how the approval was given and so you get committed to certain research models and so on. So I do get that. I just I think it’s unfortunate that there couldn’t have been some reassessment of that somewhere along the line. Because I do think it’s quite a strong [intervention]. I think it has the potential to help a lot of people (PCN manager-TC).*
○ Patients act as own controls using histories	*I’m not sure that you’re going to get better data or better results by having had this group of people that didn’t get the protocol. They could have been their own control group and then look at how many more people we could have offered it to (PCN manager-HD/TC).*
	*Or if you would want to maybe have everybody be intervention and look at their past history to be the non-intervention. Like what were they like before? What were their two years prior. Their own controls, yeah… I’m not sure that you’re going to get better data, or better results of having had this [active-control] group of people that didn’t [get the intervention] – that the protocol could have given to them (PCN provider-TC).*
○ Carefully match patients	*I think people can be their own controls or you can carefully match (PCN manager-HD/TC).*
○ Focus on qualitative rather than quantitative evidence	*I know there’s other ways of doing research. I don’t know if you want it to be more qualitative than quantitative (PCN provider-TC).*
➢ Administrative burden ▪ Examples: scheduling data collection appointments, data entry	*Initially and ongoing trying to fit into my schedule all the admin and the phone calls and the scheduling and rescheduling if people can’t make appointments and doing that on top of my other duties that I have to do has been quite a challenge (PCN provider-HD).*
	*There’s more paperwork involved than I originally anticipated – a lot more tracking of stuff. Like I’ve got binders that – I’ve got so many binders, just like wow!…For example, we have a binder for writing down all the patient appointments. And I have that in my schedule so it’s a bit tedious to have to go into another thing and write them in there… And then we have the binder with informed consent and everybody’s information page. And then we’ve got another binder for, you know? There’s just lots of tracking and paperwork and lots of stuff on the computer. I didn’t realize that it would be that much (PCN provider-HD).*
	*I feel like I was way too busy doing my own admin work to really worry about what else was going on. So I feel like, you know, there were times where the workload was ridiculously heavy. But it’s more in those recruiting stages, right? And then it kind of tapers off and you see your patients and then that’s it, right? And then you have your next wave of recruiting (PCN provider-HD).*
	*A challenge that I found was actually keeping up with [data entry in the Care Manager Tracking System (CMTS)] because I was part-time and I felt like I was double charting, right?… I found it overwhelming sometimes… But the part that always hung me up was that extra charting in the CMTS so that was where I did actually appreciate, again, the support [from the research team]– just for the quirkiness of that actual database as well as just the accountability of actually getting it done (PCN provider-TC).*
▪ Preferred passive patient recruitment and voluntary or engaged patient population	*At times I felt it was [the PCN’s] responsibility to find these participants for the study. I think it could have been pre-arranged, do you know what I mean? …Maybe doing an ad in the newspaper to get more participants, maybe going to the doctors’ offices and advertising a little bit more probably would have increased the numbers in the study. But not me doing that. I think that should have been done before. So that the numbers were already there, the participants were already volunteering to participate (PCN provider-HD).*
▪ Decreased satisfaction related to research role, specific to HEALD	*The paperwork was a bit… I mean, there’s a lot of paperwork with it, right? So that wasn’t my favourite part of it… Less paperwork and less stuff around “You gotta invite these people at this time”… Well you know, as exercise specialists, we’re all action and let’s – don’t bother me with the details kind of thing. Let’s go walking or something, you know? (PCN provider-HD).*
	*Now I understand that with, like it’s research so it had to be the way that it is and I think that that probably impacted my level of satisfaction with it. I mean I did really enjoy working in the group setting and I really think that at the end of the day just working with the people is what I enjoy (PCN provider-HD).*
	*I would consider doing [this role] again in a different capacity, with making some of the modifications that we’ve already discussed. And then in fact just doing the model. And not all the other [research] things (PCN provider-HD).*
	*Well [I was] surprised by all the admin work because it’s not something I really enjoy doing (PCN provider-HD).*

*HD* Healthy Eating and Active Living for Diabetes in Primary Care Networks (HEALD) intervention

*PCN* primary care network

*TC* TeamCare intervention

### Theme 1: eager to participate in primary care implementation research

At baseline, healthcare managers reported being eager to support and conduct implementation research in the primary care setting to prove effectiveness of interventions and to use the evidence to inform decisions around programming and sustainability (i.e., business planning):

*Number one, we’re a PCN that really does support research (PCN manager-HD/TC).*

*It will, regardless of what the results show…, it’s going to inform business planning (PCN manager-HD/TC).*

However, PCN managers and providers perceived the interventions as university-based research studies, not PCN programs, despite our efforts to promote ownership:

*If it was just brought to the PCN and it wasn’t a study and things like that, I think I would be more engaged in it because I would have adopted it as our program that’s staying… (PCN manager-TC).*

Therefore, healthcare managers and providers viewed implementation research as external to the PCNs.

### Theme 2: challenges to conducting primary care implementation research

Regardless of the initial willingness of healthcare managers to participate in research, implementation of the experimental study designs proved challenging for both interventions due to presumed effectiveness of the interventions, role conflict, and administrative burden.

#### Interventions presumed better than usual care

It appears that healthcare managers and providers had a negative perception of usual care and presumed the interventions would improve patient care at the outset of the study. Once implemented, they perceived the experimental study design as inappropriate for this stage of research because the interventions had been demonstrated *efficacious* in randomized trial settings:

*My way of thinking, we would have had a lot more buy-in, since this is already a protocol that has been proved in other places to have worked. We’re not really necessarily testing the protocol so much as the applicability to this population. Honestly, is there that much difference between Canadians and Americans as far as whether or not a protocol would work? (PCN provider-TC).*

Specific to TeamCare, healthcare managers and providers perceived poor patient engagement and retention among control group patients because they believed there was “nothing in it” for those allocated to usual care:

*[The care manager] shared with me that getting people in the [control group]… they’re just not coming back in [for data collection appointments]. And really, why should they? There’s nothing in it for them (PCN manager-TC).*

In addition, healthcare managers and providers expressed concern referring control group patients to usual care, especially for TeamCare, because patients may not access care or physicians might not refer patients to existing PCN programs and services:

*Is the client gonna really go back to the doctor? I have no control over that. He may say “Yeah I’ll go” but whether he actually goes – I’m not following him up in two weeks and saying “Hey, did you get over to your doctor yet?” (PCN provider-TC).*

Their anxiety intensified when they allocated patients they perceived as most in-need (i.e., patients who have “fallen through the cracks”, experiencing symptoms, or struggling to manage their condition) to the control group:

*This fellow yesterday, just as an example, he was 21 [on the PHQ]… his A1c was 11.1, his lipids were all elevated, blood pressure. And then I had to say, “Hmmm. Well thanks for coming out”. So that feels unethical to me ‘cause we know we could offer something more. So I have a hard time with that. This fellow yesterday, he said, “I feel like my wife and I are falling through cracks”, and here he gave a hand out and I had to say, “Well thanks for coming out. Go back to your doctor” - you know? Just didn’t seem right…Can we make him in my [intervention] group? Could I switch him? And then when he says things like, “I feel like my wife and I have fallen through the cracks”, it’s like oh here’s another one, you’re falling through the cracks ‘cause – he’s so depressed, he feels like he’s on a treadmill. He feels like giving up. He feels like jumping off a bridge. So now I have to say “Hey wait a minute, go back to your doctor” rather than saying “Hey, why don’t you come back next week and see me and we’ll talk some more about this” (PCN provider-TC).*

Indeed, we observed an imbalance in TeamCare enrollment with 95 patients assigned to the intervention group and 62 patients assigned to the control group [27]. While the groups had similar characteristics, the imbalanced numbers may reflect attempts of healthcare providers to enrol patients into the intervention group. However, this was not the case for HEALD, where the groups were more balanced numerically [27]. As a strategy to alleviate healthcare managers and providers’ concerns with the design, TeamCare study design allowed control group patients to cross over into the intervention group once they completed the control group cycle and if they were still experiencing depressive symptoms [27].

#### Role conflict

At baseline, healthcare managers and providers did not express concern implementing the “on-off” design for either intervention. However, during mid- and post-intervention interviews, they reported challenges implementing the design. This arose largely from conflict between the healthcare providers’ roles as researchers and their professional principles and training as care providers. In particular, interacting with control group patients (e.g., conducting baseline allocation and data collection) went against their commitment to provide care, problem-solve and offer services:

*It may be easier if somebody is used to research, this is just the way it goes. So you pull somebody in who’s a primary caregiver and put them into a position where they’re used to solving problems, they’re used to making suggestions. That’s what you do and then you take that away from them. It’s like you strip that ability away from them (PCN manager-HD/TC).*

Specific to TeamCare patients, the discomfort experienced by healthcare providers was extreme with many describing the experimental study design as unethical or immoral:

*We’ve all had a really hard time with those patients in the [active-control] group. And that’s so hard on our staff…That’s been very difficult for us. Hands off…And, what about ethics? Where does ethics play in something like that? Because, really, that’s not ethical (PCN manager-TC).*

For these reasons, healthcare providers recommended modifying the study designs by using patients as their own controls, carefully matching patients, or focusing on qualitative rather than quantitative evidence:

*As far as ongoing challenges, I still think that the [control] group and the design methodology has been a major barrier to success. And in the future, I have a very strong recommendation that the design methodology be reconsidered (PCN manager-HD/TC).*

#### Administrative burden

In addition to presumed intervention effectiveness and role conflict, healthcare providers identified the amount of administrative work as a challenge in implementing research in the primary care setting. Examples of burdensome administrative tasks included scheduling data collection appointments and conducting data entry for both interventions:

*I just hate it. I hate making phone calls! That’s probably my least favourite is doing the follow-up calls and leaving the same message and asking the same questions… It’s a lot of calls! Boy. It is a bit tedious after a while. So I’ll be glad when I can teach the classes and not have to do the follow-up calls anymore! (PCN provider-HD).*

*I was surprised by the amount of paperwork… it was entering it on to the paper and then into the computer as well. I don’t think there is a different way around it. It needs to be done and it’s just part of the study (PCN provider-TC).*

Healthcare providers also discussed their role in recruiting patients and preferred passive recruitment to active screening:

*I think another potential that we have promoted within the PCN is using our own [existing] patients. So when we identified a newly diagnosed [diabetic], we would screen them ourselves potentially for depression…And then the other thing I don’t think we have done is other means of recruitment. So going to the paper, looking at just the general population ‘cause there are a population of people out there that are interested in participating in studies (PCN provider-TC).*

This reflects the typical pattern of healthcare providers offering interventions to patients who seek care, rather than systematically identifying and proactively contacting targeted patients at a population level.

Specific to HEALD, exercise specialists reported dissatisfaction with their role participating in research. They indicated they would do this role again *without* the research component, because their priority is patient care, not administrative tasks:

*Working with patients is, for me, the satisfaction. Having to do the recruitment and the admin…that’s not what I want to do… I want to spend my time with the patients (PCN provider-HD).*

## Discussion

In exploring the perceptions of healthcare managers and providers involved in two implementation research studies, we found two major themes. First, healthcare managers were eager to conduct implementation research in a primary care setting. Second, regardless of willingness to participate in research, there were challenges to implementing protocols and experimental study designs for both interventions. Healthcare managers and providers presumed the interventions were better than usual care, expressed role conflict, and reported administrative burdens related to participation in research. Importantly, their perceptions of patient vulnerability and an obligation to intervene exacerbated these issues. In summary, our findings demonstrate a separation in the participants’ minds of: (1) the intervention, which is perceived as proven and valuable without further scrutiny, (2) its delivery, which is seen as unproblematic and a component of providing care, and (3) the research activities to determine its effectiveness, which are seen as unnecessary and burdensome, except among the higher echelons to justifying funding of a service.

Our findings were similar to those reported in other studies on healthcare professionals’ perspectives toward research, including valuing research [6, 33], a commitment to gathering evidence [2], and expectations of useful clinical knowledge as an outcome [6]. However, we found that, in general, healthcare managers and providers presumed the interventions were better than usual care at the outset of the study. Healthcare providers in other studies have also expressed discomfort and defensiveness related to role conflict as both researchers and providers [2, 4, 6], including moral distress [5]. They asserted their professional training and accountability took priority over research [2, 3, 5–8] potentially leading to intervening in patient care outside of the defined study protocol. As such, professional training as caregivers may compromise adherence to study protocols [4] and potentially dilute intervention effectiveness [5, 6]. For example, nurses in one study described providing increased patient-provider contacts, additional referrals, advice, and links to support groups to trial patients citing the provision of extra care was in the best interest of patients [4].

We found role conflict among healthcare providers intensified when they perceived the target patient population as vulnerable and in need of intervention based on the severity of their presenting illness. Indeed, others have highlighted challenges healthcare providers face participating in research based on the nature of the patient population. For example, physicians expressed difficulties suspending clinical judgment in assessing patient eligibility for a trial, particularly in recruiting patients they perceived as acutely unwell (e.g., young men with aggressive tumors) [2]. Similarly, nurses had “empathetic preferences” where they perceived certain interventions as less desirable for certain types of patients (e.g., seriously ill children) [6]. In our case, nurse care managers reported difficulties assigning depressed patients to the control group. This did not appear to be the case for the exercise specialists in assigning non-depressed but sedentary patients to the control group.

Lastly, we found that healthcare providers perceived participating in primary care implementation research as administratively burdensome. We found some healthcare providers did not want to recruit patients actively into the intervention studies at a population level, preferring passive recruitment of volunteers that presented for care, as is typical in day-to-day practice. However, relying on a voluntary population excludes diverse and high-risk patients (e.g., those with comorbidities, diverse ethnicities, or limited engagement with healthcare services) [21, 34]. In particular, the exercise specialists who implemented HEALD perceived administrative tasks and documentation in direct competition with providing patient care. Similarly, Spilsbury et al. [3] reported a lack of compliance with trial protocols among healthcare providers due to perceived “documentary burden”. The authors [3] found that staff perceived completion of trial paperwork as extra work that was a “tick box” procedure rather than fundamental to good patient care, which resulted in variable quality of data collected. These attitudes may have implications for practice and healthcare reform, beyond the realms of implementation research.

Our findings demonstrate a complex interplay between healthcare professionals’ presumption that interventions proven effective in similar settings are better than usual care and role conflict through the obligation to intervene in patient care based on the patient population targeted and the severity of the health condition. In addition, recruitment and systematic documentation was perceived as a burden of participating in research, rather than components of quality patient care. While we must address these issues when undertaking primary care implementation research, adapting study designs and protocols to mitigate these issues may compromise research quality (e.g., low quality data, inadequate rigour, and poor level of evidence). Acknowledging the complexities derived from our findings, we argue researchers must work to balance the conduct of rigorous implementation research while maintaining positive working relationships with staff of the healthcare organizations where this type of research must be conducted. In addition, engraining the values and practices of regular and ongoing quality management and improvement (e.g., data collection and rigorous evaluation) of health care delivery in the basic training of all healthcare providers would likely strengthen implementation research, as well as primary care reform.

Despite its strengths, our work has some limitations. First, our participants and their PCNs were relatively research naïve, and it might be that they would not perceive the same role conflicts and burdens during the next implementation study or with a different study design. Second, our findings are based on the experiences of health providers from four voluntary, non-metro PCNs that may not be representative.

## Conclusions

Healthcare managers and providers with limited practical research experience might not foresee the challenges in implementing experimental study designs and protocols in primary care settings to generate high-quality evidence. These issues become exacerbated in context of the patient population targeted and severity of their presenting illness. These findings are relevant to those designing and conducting implementation research in primary care settings, and our work and others present some possible solutions. First, it may be worthwhile to provide research training, support, and capacity building to healthcare providers that address the practical challenges of participating in primary care research [2, 3, 5–8, 33]. Second, researchers should involve healthcare providers in research planning and development a priori, including determining type of study design and eligibility criteria [2] based on assessment of the patient population and perceived severity of the health condition to address the moral obligation to provide care [5]. Third, it may be unrealistic to ask healthcare providers to balance two roles (e.g., research nurse versus clinical nurse), which have competing demands, interests, and ethics, and use of non-clinical staff may be necessary to perform research tasks [2, 5, 6] particularly with acutely unwell patient populations.

## Abbreviations

HEALD or HD, healthy eating and active living for diabetes in primary care networks; PCN, primary care network; T2D, type 2 diabetes; TC, TeamCare

## References

[1] Grimshaw JM, Eccles MP, Lavis JN, Hill SJ, Squires JE (2012). Knowledge translation of research findings. Implement Sci.

[2] Donovan JL, Paramasivan S, de Salis I, Toerien M (2014). Clear obstacles and hidden challenges: understanding recruiter perspectives in six pragmatic randomised controlled trials. Trials.

[3] Spilsbury K, Petherick E, Cullum N, Nelson A, Nixon J, Mason S (2008). The role and potential contribution of clinical research nurses to clinical trials. J Clin Nurs.

[4] Lawton J, Jenkins N, Darbyshire J, Farmer A, Holman R, Hallowell N (2012). Understanding the outcomes of multi-centre clinical trials: A qualitative study of health professional experiences and views. Soc Sci Med.

[5] Oberle K, Allen M (2006). Ethical considerations for nurses in clinical trials. Nurs Ethics.

[6] Tomlin Z, deSalis I, Toerien M, Donovan JL (2014). Patient advocacy and patient centredness in participant recruitment to randomized-controlled trials: implications for informed consent. Health Expect.

[7] Wilkes L, Cert R, Beale B (2005). Role conflict: appropriateness of a nurse researcher’s actions in the clinical field. Nurse Res.

[8] Beale B, Wilkes L (2001). Nurse researcher: always a researcher, sometimes a nurse. Collegian.

[9] Rittenhouse DR, Shortell SM (2009). The patient-centered medical home: will it stand the test of health reform?. JAMA.

[10] Reid RJ, Coleman K, Johnson EA, Fishman PA, Hsu C, Soman MP, Trescott CE, Erikson M, Larson EB (2010). The Group Health medical home at year two: cost savings, higher patient satisfaction, and less burnout for providers. Health Aff (Millwood).

[11] PCN Evolution http://pcnevolution.ca/supportwheel/Pages/OverviewofPCNE.aspx 26 July 2016.

[12] Johnson ST, Mundt C, Soprovich A, Wozniak L, Plotnikoff RC, Johnson JA (2012). Healthy eating and active living for diabetes in primary care networks (HEALD-PCN): rationale, design, and evaluation of a pragmatic controlled trial for adults with type 2 diabetes. BMC Public Health.

[13] Johnson JA, Al Sayah F, Wozniak L, Rees S, Soprovich A, Chik CL, Chue P, Florence P, Jacquier J, Lysak P (2012). Controlled trial of a collaborative primary care team model for patients with diabetes and depression: Rationale and design for a comprehensive evaluation. BMC Health Serv Res.

[14] Johnson ST, Bell GJ, McCargar LJ, Welsh RS, Bell RC (2009). Improved cardiovascular health following a progressive walking and dietary intervention for type 2 diabetes. Diabetes Obes Metab.

[15] Katon WJ, Lin EH, Von Korff M, Ciechanowski P, Ludman EJ, Young B, Peterson D, Rutter CM, McGregor M, McCulloch D (2010). Collaborative care for patients with depression and chronic illnesses. N Engl J Med.

[16] Katon WJ, Von Korff M, Lin EHB, Simon G, Ludman E, Russo J, Ciechanowski P, Walker W, Bush T (2004). The pathways study - A randomized trial of collaborative care in patients with diabetes and depression. Arch Gen Psychiatry.

[17] Johnson ST, Mundt C, Qiu W, Soprovich A, Wozniak L, Plotnikoff R, Johnson JA (2015). Increase in daily steps after an exercise specialist led lifestyle intervention for adults with type 2 diabetes in primary care: a controlled implementation trial. J Phys Act Health.

[18] Johnson JA, Al Sayah F, Rees S, Qiu W, Chik C, Chue P, Florence P, Jacquier J, Lysak P, Opgenorth A (2014). Collaborative Care vs Screening and Follow-up for Patients with Depression and Diabetes: Results of a Primary-Care Based Comparative Effectiveness Trial. Diabetes Care.

[19] Glasgow RE, Vogt TM, Boles SM (1999). Evaluating the public health impact of health promotion interventions: the RE-AIM framework. Am J Public Health.

[20] Glasgow RE, McKay HG, Piette JD, Reynolds KD (2001). The RE-AIM framework for evaluating interventions: what can it tell us about approaches to chronic illness management?. Patient Educ Couns.

[21] Glasgow RE (2003). Translating research to practice - Lessons learned, areas for improvement, and future directions. Diabetes Care.

[22] Wozniak L, Rees S, Soprovich A, Al Sayah F, Johnson ST, Majumdar SR, Johnson JA. Applying the RE-AIM framework to the Alberta's Caring for Diabetes Project: a protocol for a comprehensive evaluation of primary care quality improvement interventions. BMJ Open. 2012;2:e002099-e002109. 10.1136/bmjopen-2012-002099 PMC3488740 23103609

[23] Shediac-Rizkallah MC, Bone LR (1998). Planning for the sustainability of community-based health programs: conceptual frameworks and future directions for research, practice and policy. Health Educ Res.

[24] Garfield SA, Malozowski S, Chin MH, Narayan KM, Glasgow RE, Green LW, Hiss RG, Krumholz HM (2003). Considerations for diabetes translational research in real-world settings. Diabetes Care.

[25] Proctor E, Silmere H, Raghavan R, Hovmand P, Aarons G, Bunger A, Griffey R, Hensley M (2011). Outcomes for implementation research: conceptual distinctions, measurement challenges, and research agenda. Adm Policy Ment Health.

[26] Majumdar SR, Rowe BH, Folk D, Johnson JA, Holroyd BH, Morrish DW, Maksymowych WP, Steiner IP, Harley CH, Wirzba BJ (2004). A controlled trial to increase detection and treatment of osteoporosis in older patients with a wrist fracture. Ann Intern Med.

[27] Mathe N, Johnson ST, Wozniak L, Majumdar SR, Johnson JA (2015). Alternation as a form of allocation for quality improvement studies in primary health care settings: the on-off study design. Trials.

[28] Morse JM, Field PA (1995). Qualitative Research Methods for Health Professionals.

[29] Mayan MJ (2009). Essentials of qualitative inquiry.

[30] Morse JM, Barret M, Mayan M, Olson K, Spier J (2002). Verification Strategies for Establishing Reliability and Validity in Qualitative Research. Int J Qual Methods.

[31] Guba EG, Lincoln YS (1989). Fourth Generation Evaluation.

[32] Nvivo 10 [http://www.qsrinternational.com/product] 26 July 2016.

[33] Jones A, Burgess TA, Farmer EA, Fuller J, Stocks NP, Taylor JE, Waters RL (2003). Building research capacity. An exploratory model of GPs’ training needs and barriers to research involvement. Aust Fam Physician.

[34] Wozniak L, Soprovich A, Rees S, Johnson ST, Majumdar SR, Johnson JA (2015). Challenges in identifying patients with type 2 diabetes for quality-improvement interventions in primary care settings and the importance of valid disease registries. Can J Diabetes.

